# Reversible high blood CEA and CA19-9 concentrations in a diabetic patient

**DOI:** 10.3402/ljm.v7i0.19572

**Published:** 2012-10-24

**Authors:** Pei-Chi Chen, Hong-Da Lin

**Affiliations:** Division of Endocrinology and Metabolism, Shin Kong Wu Ho-Su Memorial Hospital, Taipei, Taiwan

We observed a 31-year-old male suffering from general malaise and body weight loss for almost 6 months. He came to our hospital for examination, and the reports showed recently diagnosed diabetes (fasting plasma glucose 309 mg/dl, HbA1C 14.9%) with elevated carcinoembryonic antigen (CEA) 8.9 ng/ml (normal range 0–5 ng/ml) and carbohydrate antigen 19-9 (CA19-9) 123 U/ml (normal range 0–37 U/ml). Abdominal computed tomography (CT) revealed faint hypodensity at pancreatic head with mild dilatation of the main pancreatic duct. Positron emission tomography (PET) scan was then arranged to rule out malignancy and the report showed no abnormal fludeoxyglucose (FDG) uptake over the pancreatic head. After 3 months of anti-diabetic drugs treatment with lifestyle modification, his HbA1C fell to 6.8%. At the same time, the tumor markers also declined to normal (CEA 3.8 ng/ml, CA19-9 26.58 U/ml).

CEA and CA 19-9 are closely related with gastrointestinal cancers, although they are not suggested for cancer screening (1). Pancreatic cancer may cause diabetes by destroying islet cells, inducing pancreas inflammation, or causing peripheral resistance to insulin. Therefore, diabetes mellitus may be the first manifestation of pancreatic cancer (2, 3). Lack of family history of diabetes, absence of obesity, rapid deterioration of hyperglycemia, or elevation of tumor markers or amylase, may present clues for the earlier diagnosis of pancreatic cancer (4, 5). Guo et al. (6) suggested that new-onset diabetes combined with CEA ≥ 5 ng/ml and/or CA 19-9 ≥ 500 U/ml might be regarded as a tool to screen early pancreatic cancer.

Elevated CA19-9 in patients with various benign diseases, such as chronic liver disease, pancreatitis, interstitial pulmonary disease, and endometriosis has been reported in the literature (7). Diabetes mellitus is also one of the benign diseases related to CA19-9 elevation. A few studies (8–11) demonstrated that blood CA19-9 level was positively correlated with glycemic control and HbA1c level. They also reported that decrease of CA19-9 paralleled the improvement of glycemic control. To prevent unnecessary further procedures, Kamile et al. (8) proposed a higher CA19-9 cutoff value of 57.14 U/ml instead of the usual upper limit of 37 U/ml for patients with diabetes.

The histology of pancreatic islets from type 2 diabetic patients was known to be associated with an inflammatory process, which also involved the exocrine pancreas (12–14). Mildly elevated blood lipase and amylase associated with faint hypodensity at pancreatic head in a CT image suggested the presence of a subclinical, mild form of pancreatitis, which may be responsible for the temporary elevation of blood CEA and CA19-9 concentration in this new-onset diabetes.

Although serum tumor markers are infrequently determined in diabetic patients, blood CEA and CA19-9 are occasionally measured for other reasons. We should be very careful to differentiate between the benign and malignant etiologies of the elevated tumor markers in diabetes.

*Pei-Chi Chen* Division of Endocrinology and Metabolism Shin Kong Wu Ho-Su Memorial Hospital Taipei, Taiwan*Hong-Da Lin* Division of Endocrinology and Metabolism Shin Kong Wu Ho-Su Memorial Hospital Taipei, TaiwanDivision of Endocrinology and Metabolism Taipei Veterans General Hospital Taipei, Taiwan Email: M007455@ms.skh.org.tw
